# Studies on *N*-Ethyl-*N*-nitrosourea Mutagenesis in BALB/c Mice

**DOI:** 10.5487/TR.2008.24.1.059

**Published:** 2008-03-01

**Authors:** Kyu-Hyuk Cho, Jae-Woo Cho, Chang-Woo Song

**Affiliations:** Department of Research & Development, Korea Institute of Toxicology, Korea Research Institute of Chemical Technology, P.O BOX 123, Yuseong, Daejeon, 305-343 Korea

**Keywords:** ENU mutagenesis, Mutant, Dose, ECPS, BLAB/cAnN mice

## Abstract

N-ethyl-N-nitrosoures (ENU) is effective in inducing hypermorphic mutation as well as hypomorphic and antimorphic mutations. Therefore, this mutagen is used to the production of mutant in the mice. In order to perform an effective ENU mutagenesis using BALB/cAnN mice, determination of optimal dosage and dosage regimen of ENU is necessary. And this study tried to develop a suitable screening method and searched for novel and various mutants as model animals in phenotypedriven ENU mutagenesis. We have carried out dosage regimen for mutagenizing dose of 200 mg/kg ENU in the BALB/c mice. Total screened mice were 30,133. As the results of Esaki and Cho’s Phenotype Screening, we got 2,516 phenotypic and behavior abnormalities in G_1_, G_2_ and G_3_ mice. One hundred thirty five G1 phenodeviants were tested for inheritance and 16 dominant mutants were discovered. Forty two recessive mutants were also found in tested 201 micropedigrees. Early-onset mutant mice included the dysmorphology of face, eye, tail, limb, skin, and foot and abnormal behavior like circling, swimming, head tossing, stiff-walking, high cholesterol level, and tremor etc. In this study we could effectively screen G3 recessive mutants. The frequent and concise early-onset screening before weaning will be available for ENU mutagenesis.

## INTRODUCTION

The reports of the DNA sequence of the human genome and the mouse genome, the first phase of the Human Genome Project is complete (Venter *et al.*, 2001; Waterston *et al.*, 2002). The sequence information, however, does not reveal the functions of most genes encoded by the genome. Attempts have been made to elucidate the functions of human genes using various methods such as overexpression of certain genes (Wagner *et al.*, 2003; Sawyer *et al.*, 2003). The establishment of embryonic stem (ES) cells combined with homologous recombination made it possible to delete a specific gene or DNA segment (Dominguez-Bendala *et al.*, 2003; Zwaka and Thomson *et al.*, 2003). This technique, albeit many shortcomings, has been proven to be powerful in defining the function of human genes *in vivo*. Improved and modified methodology of gene targeting began to utilized for mass production of mutants in the era of 'post-Human Genome Project'.

Phenotype-driven approach, on the contrary to the genotype-driven approaches such as gene targeting and gene trapping, gains strength in defining gene functions and 'ENU (ethylnitrosourea) mutagenesis' is proven to be the most powerful method for mass production of mouse mutants (Nolan *et al.*, 2000; Hrabe de Angelis *et al.*, 2000). ENU has been originally used as positive control substance of genotoxicity studies (Shibuya and Morimoto, 1993). ENU can transfer its ethyl group to oxygen or nitrogen radicals in DNA, resulting in mispairing and base-pair substitution if not repaired. The highest mutation rates occur in pre-meiotic spermatogonial stem cells, with single locus mutation frequencies of $6\sim1.5 \times 10^{-3}$ equivalent to obtaining a mutation in a single gene of choice in one out of every 175~655 gametes screened. Because it is a point mutagen, ENU can induce many different types of allele. Loss-of-function mutations, viable hypomorphs of lethal complementation groups, antimorphs and gain-of-function mutations have been isolated in mouse mutagenesis screens (Justice *et al.*, 1999; Weber *et al.*, 2000). Although this is initially a disadvantage with respect to the cloning of the responsible gene(s), the availability of point mutations will be very important for a more detailed functional analysis of many genes. Furthermore, the advances that are currently made in the field of genomics, particularly the production of high resolution genetic, physical and transcript maps will reduce the difficulties inherent in the cloning of the genes mutated after ENU treatment.

For these reasons, we carry out a systematic production of mouse mutants by ENU. Already, we reported the effect on the reproduction of BALB/cAnN male mice after ENU treatment. The observation frequency of phenodeviants and the kinds of novel mutants were reported in this paper.

## MATERIALDS AND METHODS

***Mice and husbandry.*** BALB/cAnN male and female mice were used. All mice were bred at Korea Research Institute of Chemical Technology/Korea Institute of Toxicology. They were reared in the SPF animal room controlled at 23 ± 3°C of the temperature and 50 ± 10% of the relative humidity, and in conformity with the *Guide for the Care and Use of Laboratory Animals* (NIH). All animal experiments were carried out in accordance with the *Guidelines for Animal Experimentation* and *Institutional Animal Care and Use*. Each male was housed individually during ENU administration and until to start mating. Table 7 shows the information of experimented mice.

***Administration of ENU.*** ENU (*N*-ethyl-*N*-nitrosourea, Sigma, St. Louis, MO: Lot No. 3385) was dissolved to 1 g/100 ml in 1/15 M phosphate buffer (pH 6.0) immediately before administration. Male mice were weighted and injected intraperitoneally at fractionated doses: 0, 75, 100, 125 and 150 mg/kg mouse body weight for two or four consecutive weeks. All injections were completed within one hour after the ENU was dissolved.

***Clinical signs, body weight and survival rate.*** Clinical signs were monitored every day until the final injection (14th day), and then once a week thereafter. The body weight of each mouse was weighted using an automatic electronic balance (Sartorious Co., Germany) at the initiation of treatment, once a week. Statistical analysis of body weights were evaluated by ANOVA (one way analysis of variance) multiple comparison test (Turkey test) using GraphPad Instate (V2.05a). The level of significance was taken a P < 0.05. Survival rate was observed till 30th week after ENU administration. Dead animals were subjected to autopsy and fixed in formaldehyde.

***Mating schedule.*** We introduced the strategy of mating experiment (Fig. 1). The mating was conducted

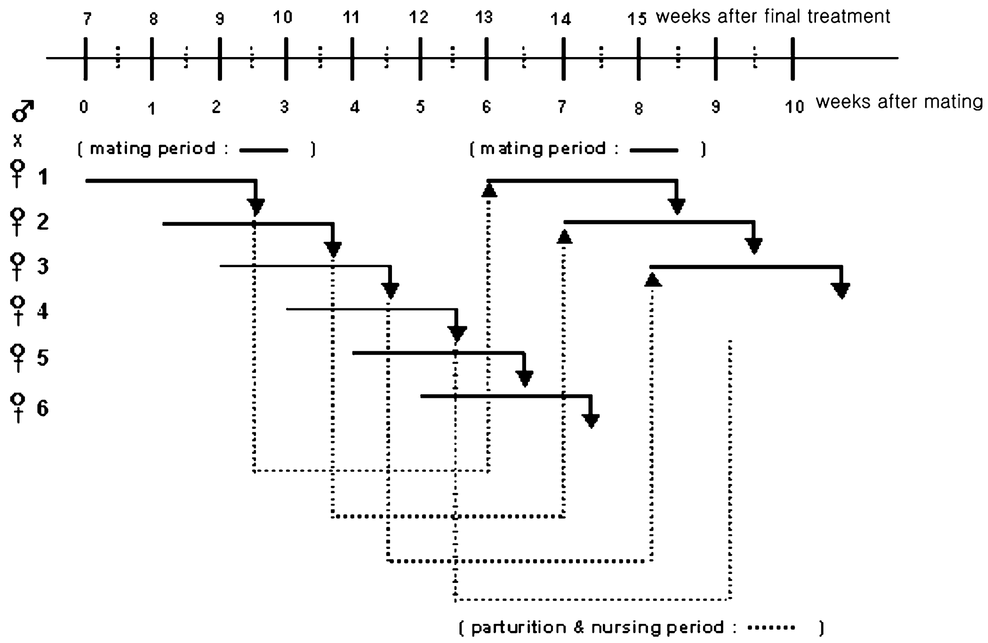

**Fig. 1.** Strategy of mating experiment. After 7 weeks of the final injection, each male was mated to female, and added a new female on each week. Each female was moved into the individual breeding cage after each 2.5 weeks period of mating for her parturition and nursing. After weaning of young mice or on the 6th week after the previous mating, each female was moved back to the same male of the previous mating to start the cycle over again.

for 11, 42, 302, 30, 10, and 18 males in each 0, 150, 200, 250, 300, and 400 mg/kg group, respectively. After 6 weeks of the final injection, each male was mated to a female, and added a new female on each week. Each female was moved into the individual breeding cage after each 2.5 weeks period of mating for her parturition and nursing. After weaning of young mice or on the 6th week after the previous mating, each female was moved back to the same male of the previous mating to start the cycle over again.

***Average fertile regain period, total litter size and average litter size.*** The fertile regain period was recorded based on the time period at which mating and fertilization were established. 20 days of gestation period were subtracted from the birth day of $G_1$, $G_2$, and $G_3$ offspring derived from EUN-treated males and wild type females. The average fertile regain period and its scope were investigated at each dose. In addition, the total litter size and average litter size were recorded. The total litter size was calculated by counting all fetuses at birth, and the average litter size was calculated by dividing the total litter size by the total number of births.

***ECPS protocol.*** For the purpose of primary for early-onset phenodeviants, ECPS (Esaki and Cho's phenotype screening) protocol was established and applied to ENU mutagenesis program by KIT/KRICT (Korea Institute of Toxicology, Korea Research Institute Chemical of Technology). This observation battery about behavior, reflex, growth, morphology was based on Irwin screening items for pharmacological purposes (Cho *et al.*, 2002). After observation, abnormal, normal, or content were recorded on observation card. Until the mice were weaned, observations were made 5 times at birth, 4th, 7th, 14th, 21st day old about those items in the Perspex standard mouse cage. Body weight of mice was record at 7th, 14th, 21st day old with observation. This protocol was used in all screening for dominant and recessive mutants.

***Blood screen.*** To develop the mutants with the abnormal lipid profile of total cholesterol (TC), triglyceride, HDL-cholesterol (HDL-C), and LDL-cholesterol (LDL-C), the serum of 811 heads were analyzed when they were 8~17 weeks old. Approximately 130 μl serum was collected from the ophthalmic venous plexus per one mouse. Each samples measured using an autoanalyzer (Shimadzu CL-7200, Shimadzu Co., Japan).

***Inheritance test and mutant line maintenance.*** As for dominant mutations, a screened phenodeviant was mated to a wild type female to produce $G_2$ generation. If 50% of the character identical to $G_1$ was displayed, a mutation line was considered as confirmed. The screen for recessive mutations involved mating a $G_1$ heterozygote type and heterozygote type $G_3$ to produce $G_4$ generation. If 25% per parity was displayed in $G_4$ generation, a mutation line was considered as confirmed. A dominant mutant line was maintained by mating a mutant mouse and a wild type mouse, and a recessive

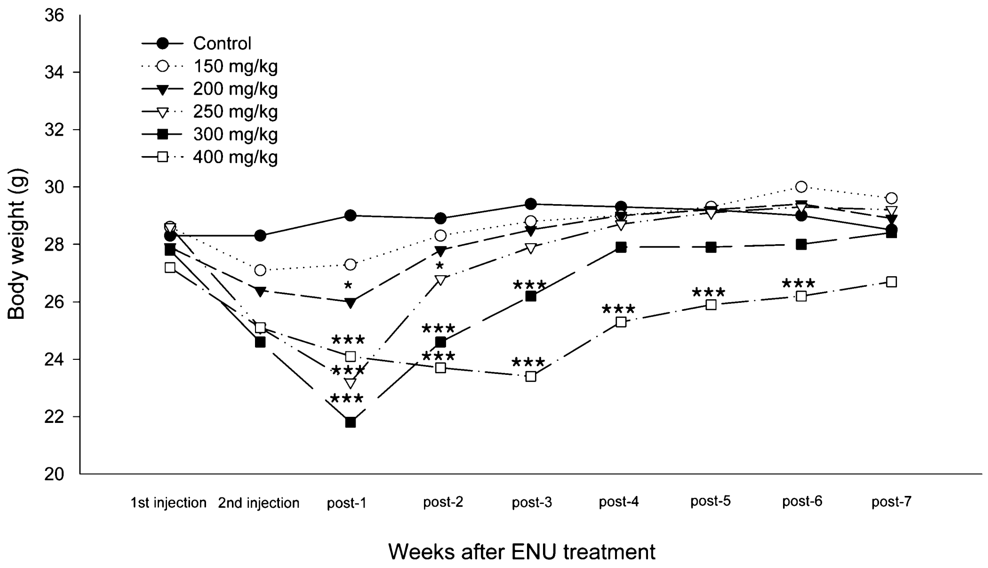

**Fig. 2.** Changes of body weight with ENU treatment. *: Significantly different compared with data of the control group ($p < 0.05$). ***: Significantly different compared with data of the control group ($p < 0.001$).

mutant line was maintained by mating a female heterozygote and a male heterozygote.

## RESULTS

Body weight changes in mice following ENU administration are shown in Fig. 2. When body weights measured before and after ENU administration were compared, a significant decrease in body weight was noted at 150 mg/kg (week 2nd injection to 1, $p < 0.01$ and 0.05), 200 mg/kg (week 2nd injection to 1, $p < 0.001$), 250 mg/kg (week 2nd injection to 2, $p < 0.001$ or 0.05), 300 mg/kg (week 2nd injection to 2, $p < 0.01$ or 0.001) and 400 mg/kg (week 1 to 3, $p < 0.05$ or 0.01), and a significant increase in body weight was noted at 150 mg/kg (week 6, $p < 0.05$) and 200 mg/kg (week 3 to 7, $p < 0.001$). A significant decrease in body weight was observed at 200 mg/kg (week 1, $p < 0.001$), 250 mg/kg (week 1~2, $p < 0.001$ or $P < 0.05$), 300 mg/kg (week 1~3, $p < 0.001$) and 400 mg/kg (week 1~6, $p < 0.001$) as compared to the control group. Clinical signs observed in mice immediately following EUN administration are shown in Fig. 3. Irregular respiration, decreased locomotor activity and piloerectin were observed in mice immediately following ENU administration. However, recovery to normal level was noted within 2 days in each case. Gross findings at necropsy in death mice after ENU administration revealed splenomegaly, swelling of thymus and atrophy of the testis (Fig. 4).

The lethal rates and sterility periods in mice at the weekly age of up to 30 weeks following ENU administration were shown in Table 1. The lethal rates until 30 weeks following ENU administration were 0%, 13%, 11~30%, 30~40%, 40%, and 72% as doses of 0 mg/kg (vehicle control), 150 mg/kg, 200 mg/kg, 250 mg/kg, 300 mg/kg, and 400 mg/kg, respectively. Fertility was established immediately following mating in the vehicle control group. Temporary sterility periods observed were: 8~22 weeks, 9~29 weeks, and 9~25 weeks as doses of 150 mg/kg, 200 mg/kg, and 250 mg/kg, respectively. However, at 300 mg/kg and 400 mg/kg, fertile ability was not regained for 30 weeks following ENU administration.

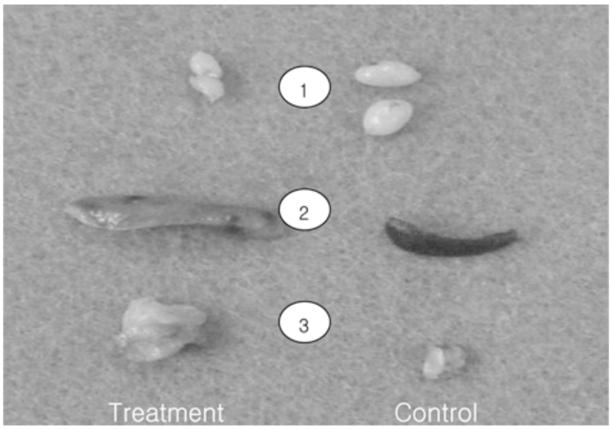

**Fig. 4.** Gross finding of affected organs in mortal animal after ENU treatment. Severe atrophy in testis ① Swelling in spleen ② and thymus ③ were observed.

The regain of fertility rates in male mice administered ENU are shown in Table 1. The period required for regaining fertility was 12~16 weeks, 14~20 weeks, and 16~18 weeks at doses of 150 mg/kg, 200 mg/kg, and 250 mg/kg, respectively. The administration of ENU resulted in gestation rates of 100%, 84~91%, 31~93%, 20~90% as doses of 0 mg/kg (vehicle control), 150 mg/kg, 200 mg/kg, and 250 mg/kg, respectively. The lethal rates in males which regained fertile ability were 6~14%, 5~26%, and 0~50% at doses of 150 mg/kg, 200 mg/kg, and 250 mg/kg, respectively. The total litter sizes and average litter sizes derived from males receiving ENU and normal females are shown in Table 2. The average litter size of $G_1$ were 5.9, 5.4~4.7, 1.9~5.1, and 1.0~3.8 at doses of 0 mg/kg (vehicle control), 150 mg/

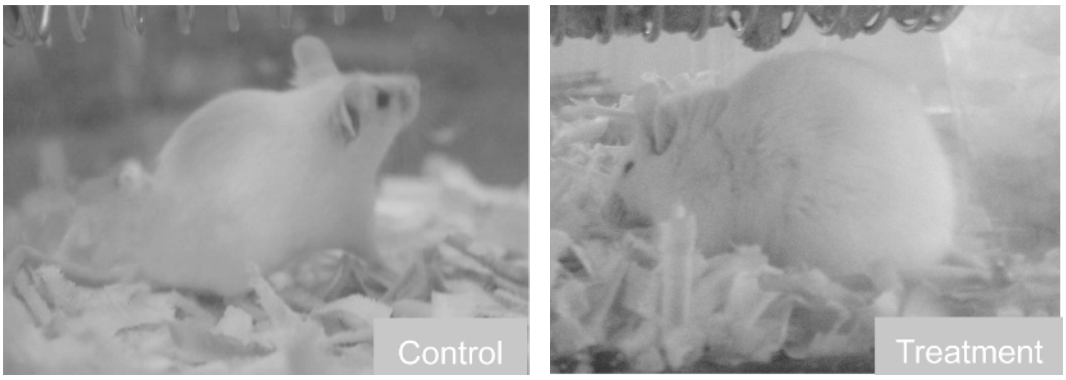

**Fig. 3.** Clinical sign after ENU treatment at 1 hour (drowsy & bristling of hair).

**Table 1.** Dose-related mutagenic responses to different doses of *N*-ethyl-*N*-nitrosourea

| Group | Dose (mg/kg) | No. of males | No. of females | No. males died (%) | Average fertile regain days (Range) | No. males fertile (%) | No. males fertile died (%) |
|---|---|---|---|---|---|---|---|
| Control | 0 | 11 | 44 | 0 (0) | 50 (42-63) | 11 (100) | 0 (0) |
| A150 | 2 × 75 | 23 | 138 | 3 (13) | 84 (56-133) | 21 (91) | 3 (14) |
| H150 | 2 × 75 | 19 | 104 | 2 (13) | 110 (77-154) | 16 (84) | 1 (6) |
| A200 | 2 × 100 | 23 | 132 | 2 (11) | 98 (63-147) | 18 (78) | 1 (6) |
| B200 | 2 × 100 | 55 | 264 | 8 (15) | 99 (70-182) | 47 (85) | 5 (11) |
| D200 | 2 × 100 | 61 | 271 | 16 (26) | 122 (98-140) | 19 (31) | 5 (26) |
| E200 | 2 × 100 | 19 | 227 | 5 (26) | 100 (91-126) | 9 (47) | 2 (22) |
| F200 | 2 × 100 | 69 | 320 | 18 (26) | 124 (77-189) | 39 (56) | 9 (23) |
| H200 | 2 × 100 | 10 | 45 | 3 (30) | 138 (105-182) | 7 (70) | 1 (14) |
| J200 | 2 × 100 | 40 | 204 | 11 (28) | 114 (91-203) | 37 (93) | 9 (24) |
| K200 | 2 × 100 | 25 | 112 | 3 (12) | 105 (84-154) | 19 (76) | 1 (5) |
| H250 | 2 × 125 | 10 | 39 | 3 (30) | 112 (63-140) | 3 (30) | 0 (0) |
| J250 | 2 × 125 | 10 | 46 | 4 (40) | 124 (105-175) | 9 (90) | 3 (33) |
| K250 | 2 × 125 | 10 | 45 | 3 (30) | 119 (-) | 2 (20) | 1 (50) |
| K300 | 2 × 150 | 10 | 20 | 4 (40) | ND | 0 (0) | 0 |
| E400 | 4 × 100 | 18 | 108 | 13 (72) | ND | 0 (0) | 0 |

ND; no data.

**Table 2.** Affect of total and average litter size to different doses of *N*-ethyl-*N*-nitrosourea

| Group | Total litter size (heads) | | | Average litter size (heads) | | |
|---|---|---|---|---|---|---|
| | $G_1$ | $G_2$ | $G_3$ | $G_1$ | $G_2$ | $G_3$ |
| Control | 1,118 | ND | ND | 5.9 | ND | ND |
| A150 | 970 | 473 | 1,202 | 4.7 | 5.4 | 4.3 |
| H150 | 595 | 468 | ND | 5.4 | 5.4 | ND |
| A200 | 1,011 | 1,594 | 2,586 | 4.4 | 4.9 | 4.2 |
| B200 | 1,433 | 2,206 | 4,364 | 4.0 | 5.7 | 4.8 |
| D200 | 197 | 897 | 1,142 | 2.6 | 6.1 | 4.1 |
| E200 | 70 | 1,870 | 2,202 | 1.9 | 5.4 | 4.6 |
| F200 | 514 | 1,350 | 1,259 | 3.7 | 5.1 | 4.5 |
| H200 | 76 | 137 | 136 | 3.5 | 4.9 | 4.5 |
| J200 | 1,224 | ND | ND | 5.1 | 5.3 | ND |
| K200 | 380 | ND | ND | 4.3 | ND | ND |
| H250 | 20 | ND | ND | 3.3 | ND | ND |
| J250 | 123 | 277 | 237 | 3.8 | 6.2 | 4.7 |
| K250 | 2 | ND | ND | 1.0 | ND | ND |
| K300 | ND | ND | ND | ND | ND | ND |
| E400 | ND | ND | ND | ND | ND | ND |

ND; no data.

**Table 3.** Summary of phenodeviant incidences and rates identified in each treatment group

| Group | No. phenodeviants (heads) | | | Phenodeviant incidence (%) | | |
|---|---|---|---|---|---|---|
| | $G_1$ | $G_2$ | $G_3$ | $G_1$ | $G_2$ | $G_3$ |
| A150 | 79 | 55 | 102 | 8 | 12 | 8 |
| H150 | 44 | 37 | ND | 7 | 8 | ND |
| A200 | 84 | 230 | 279 | 8 | 14 | 11 |
| B200 | 145 | 94 | 343 | 10 | 4 | 8 |
| D200 | 12 | 66 | 155 | 6 | 7 | 14 |
| E200 | 5 | 91 | 275 | 7 | 5 | 12 |
| F200 | 57 | 51 | 99 | 11 | 4 | 8 |
| H200 | 4 | 1 | 7 | 5 | 1 | 5 |
| J200 | 75 | 44 | 23 | 6 | 19 | ND |
| K200 | 19 | ND | ND | 5 | ND | ND |
| H250 | 1 | ND | ND | 1 | ND | ND |
| J250 | 8 | 7 | 24 | 7 | 3 | 10 |
| K250 | ND | ND | ND | ND | ND | ND |
| K300 | ND | ND | ND | ND | ND | ND |
| E400 | ND | ND | ND | ND | ND | ND |

ND; no data.

kg, 200 mg/kg, and 250 mg/kg, respectively.

The numbers of phenodeviant incidences, incidence rates, and number of animals per parameter in $G_1$, $G_2$, and $G_3$ mice are summarized in Table 3, and Table 4. The total numbers of G1 phenodeviant incidences were 44~79, 4~145, and 1~8 at doses of 150 mg/kg, 200 mg/kg, and 250 mg/kg, respectively. The phenodeviant incidence rates were 7~8%, 5~11%, and 1~7% at doses of 150 mg/kg, 200 mg/kg, and 250 mg/kg, respectively. The numbers of phenodeviant per parameter are as follows: 881 for tail (loss, bent, less, kink, tip bleeding etc), 582 for small body size, 397 for eyes (close, cataract, abnormal cornea, small etc), 98 for hair, 92 for craniofacial, 37 for tremor, 27 for head tosser, 19 for ataxia, 18 for circling, and 16 for acoustic startle. The phenodeviant incidence rate of biochemistry examination was screened 9.6% (data not shown).

The dominant and recessive mutation rates are summarized in Table 5. The dominant and recessive mutation rates were 6% (inheritance test, 36 heads) and 13% (inheritance test, 16 micropedigree), respectively at 150 mg/kg and 15% (inheritance test, 36 litters) and 23% (inheritance test, 16 micropedigree), respectively at 200 mg/kg. No mutations were confirmed at 250 mg/kg

**Table 4.** Numbers of abnormal phenotypes observed in each generation and treatment group

| Group | Items Gen.* | Morphology: Head | Hair | Limbs & toes | Tail | Eye | Body weight | Others | Behavior: Acoustic startle | Tremor | Circling | Ataxia | Head toss | Others |
|---|---|---|---|---|---|---|---|---|---|---|---|---|---|---|
| A150 | $G_1$ | 0 | 2 | 7 | 57 | 5 | 8 | 0 | 0 | 0 | 0 | 0 | 0 | 0 |
| | $G_2$ | 0 | 0 | 3 | 27 | 17 | 5 | 0 | 1 | 0 | 0 | 1 | 0 | 1 |
| | $G_3$ | 1 | 3 | 1 | 49 | 18 | 12 | 0 | 2 | 0 | 0 | 0 | 5 | 11 |
| H150 | $G_1$ | 0 | 3 | 5 | 10 | 11 | 12 | 1 | 0 | 0 | 0 | 0 | 0 | 2 |
| | $G_2$ | 0 | 0 | 25 | 3 | 0 | 9 | 0 | 0 | 0 | 0 | 0 | 0 | 0 |
| A200 | $G_1$ | 0 | 4 | 5 | 50 | 8 | 12 | 0 | 0 | 0 | 0 | 0 | 0 | 5 |
| | $G_2$ | 1 | 0 | 15 | 116 | 44 | 18 | 0 | 0 | 36 | 0 | 0 | 0 | 0 |
| | $G_3$ | 4 | 2 | 12 | 118 | 78 | 32 | 0 | 12 | 0 | 7 | 7 | 4 | 3 |
| B200 | $G_1$ | 21 | 14 | 2 | 56 | 15 | 15 | 4 | 0 | 0 | 0 | 0 | 1 | 17 |
| | $G_2$ | 12 | 3 | 2 | 28 | 13 | 28 | 1 | 0 | 0 | 1 | 0 | 0 | 6 |
| | $G_3$ | 26 | 30 | 10 | 57 | 31 | 132 | 4 | 0 | 0 | 1 | 0 | 10 | 42 |
| D200 | $G_1$ | 1 | 1 | 0 | 3 | 3 | 2 | 1 | 0 | 0 | 0 | 0 | 0 | 1 |
| | $G_2$ | 0 | 0 | 6 | 10 | 4 | 40 | 0 | 0 | 0 | 0 | 0 | 0 | 6 |
| | $G_3$ | 0 | 10 | 13 | 32 | 22 | 54 | 3 | 0 | 0 | 2 | 0 | 1 | 18 |
| E200 | $G_1$ | 0 | 0 | 0 | 3 | 0 | 1 | 0 | 0 | 0 | 1 | 0 | 0 | 0 |
| | $G_2$ | 0 | 3 | 4 | 57 | 24 | 3 | 0 | 0 | 0 | 0 | 0 | 0 | 0 |
| | $G_3$ | 26 | 0 | 15 | 107 | 60 | 41 | 0 | 0 | 0 | 4 | 11 | 1 | 10 |
| F200 | $G_1$ | 0 | 4 | 6 | 21 | 4 | 16 | 1 | 0 | 0 | 2 | 0 | 0 | 3 |
| | $G_2$ | 0 | 2 | 9 | 4 | 3 | 27 | 2 | 0 | 1 | 0 | 0 | 0 | 3 |
| | $G_3$ | 0 | 10 | 11 | 25 | 11 | 15 | 3 | 0 | 0 | 0 | 0 | 4 | 20 |
| H200 | $G_1$ | 0 | 1 | 0 | 1 | 1 | 0 | 0 | 1 | 0 | 0 | 0 | 0 | 0 |
| | $G_2$ | 0 | 0 | 0 | 0 | 0 | 1 | 0 | 0 | 0 | 0 | 0 | 0 | 0 |
| | $G_3$ | 0 | 0 | 0 | 2 | 1 | 4 | 0 | 0 | 0 | 0 | 0 | 0 | 0 |
| J200 | $G_1$ | 0 | 3 | 2 | 19 | 11 | 32 | 2 | 0 | 0 | 0 | 0 | 1 | 5 |
| | $G_2$ | 0 | 0 | 0 | 14 | 0 | 28 | 2 | 0 | 0 | 0 | 0 | 0 | 0 |
| | $G_3$ | 0 | 0 | 0 | 2 | 1 | 15 | 5 | 0 | 0 | 0 | 0 | 0 | 0 |
| K200 | $G_1$ | 0 | 0 | 0 | 3 | 7 | 4 | 0 | 0 | 0 | 0 | 0 | 0 | 5 |
| H250 | $G_1$ | 0 | 0 | 0 | 0 | 0 | 1 | 0 | 0 | 0 | 0 | 0 | 0 | 0 |
| J250 | $G_1$ | 0 | 3 | 0 | 3 | 1 | 0 | 0 | 0 | 0 | 0 | 0 | 0 | 0 |
| | $G_2$ | 0 | 0 | 0 | 2 | 3 | 0 | 0 | 0 | 0 | 0 | 0 | 0 | 0 |
| | $G_3$ | 0 | 0 | 0 | 2 | 1 | 15 | 6 | 0 | 0 | 0 | 0 | 0 | 0 |

*; Generation.

when the inheritance test of micropedigree for 3 dominants and 9 recessives were performed. The list of mutations for each test batch is shown in Table 6. The mutation phenotype for each parameter are as follows: 13 lines for behaviors, 12 lines for body type, 8 lines each for tail, face, and blood, 6 lines for hair, 2 lines for eyes, and 1 line for digit & toe. A few mutants with visible phenotypes showed in the Korea ENU mutagenesis program (Fig. 5).

## DISCUSSION

A change in body weight is the most efficient and effective indicator in evaluating physiological changes associated with various test articles in a living organism. In this study, a transient change in body weight was observed in mice administered ENU at 200 mg/kg or greater. This finding corresponded with a previous ENU mutagenesis study in which decreased body weight in mice was reported (Justice *et al.*, 2000). Interestingly, in this study the dose groups were divided into two categories: one category showing decreased body weight following ENU administration (250, 300, and 400 mg/kg) and the other category showing increased body weight following ENU administration (200 mg/kg). The former doses were considered to be reversible doses from the toxic effects of ENU, whereas the latter dose was considered to exceed the tolerated dose of the toxicity of ENU. Moreover, gross findings at necropsy on animals found dead revealed atrophy of testis, splenom-

**Table 5.** Number of mutant and inheritance test performed to each group

| Group | Inheritance test | | No. dominant mutants (%) | No. recessive mutants (%) |
|---|---|---|---|---|
| | No. male & female (for dominant) | No. micropedigree (for recessive) | | |
| A150 | 27 | 16 | 0 (0) | 2 (13) |
| H150 | 9 | 0 | 2 (22) | ND |
| A200 | 37 | 34 | 4 (11) | 10 (29) |
| B200 | 21 | 52 | 4 (19) | 10 (19) |
| D200 | 0 | 30 | ND | 4 (13) |
| E200 | 5 | 25 | 0 (0) | 15 (60) |
| F200 | 22 | 30 | 4 (18) | 1 (3) |
| H200 | 0 | 5 | ND | 0 (0) |
| J200 | 11 | 0 | 2 (18) | ND |
| K200 | ND | ND | ND | ND |
| H250 | ND | ND | ND | ND |
| J250 | 3 | 9 | 0 (0) | 0 (0) |
| K250 | ND | ND | ND | ND |
| K300 | ND | ND | ND | ND |
| E400 | ND | ND | ND | ND |

ND; no data.

**Table 6.** Mutant list derived to each *N*-ethyl-*N*-nitrosourea treatment group

| Group | Dose (mg/kg) | Mutant | | | | | |
|---|---|---|---|---|---|---|---|
| | | No. | Phenotype | Inheritance type | No. | Phenotype | Inheritance type |
| A150 | 2 × 75 | ADM16 | Microgenia | recessive | ADM03 | Cataract | recessive |
| H150 | 2 × 75 | HDM07 | SBS* | dominant | HDM04 | Limb grasping | dominant |
| A200 | 2 × 100 | AM09 | Microgenia | recessive | AM11 | Microdactylia | recessive |
| | | AM17 | Cataract | recessive | AM17 | SBS + cataract | recessive |
| | | ADM09 | Club foot | recessive | ADM22 | Short face | recessive |
| | | ADF03 | Microgenia | recessive | ADF16 | Short face | recessive |
| | | AM20 | SBS | recessive | AM21 | SBS+bent tail | recessive |
| | | AM15 | Circling | dominant | ADM22 | Bent tail | dominant |
| | | ADM29 | Tremor | dominant | ADF16 | Bent tail | dominant |
| B200 | 2 × 100 | BM15 | Nude like | recessive | BM24 | SBS | recessive |
| | | BM36 | Lamb wool | recessive | BM42 | SBS | recessive |
| | | BM52 | SBS | recessive | SB01 | Low locomotion | recessive |
| | | SB04 | Head nod | recessive | BBM05 | High cholesterol | recessive |
| | | BBM09 | High cholesterol | recessive | BBM14 | High cholesterol | recessive |
| | | BD20 | Hair poor | dominant | BD21 | Bent tail | dominant |
| | | SB02 | Deafness | dominant | SB03 | High startle | dominant |
| D200 | 2 × 100 | DM10 | Dystrophy | recessive | BDM01 | High cholesterol | recessive |
| | | BDM02 | High cholesterol | recessive | BDM05 | High cholesterol | recessive |
| | | BDM08 | High cholesterol | recessive | | | |
| E200 | 2×100 | EM03 | Short face | recessive | EM04 | Tremor | recessive |
| | | EM04 | Head tosser | recessive | EM05 | Wiggle | recessive |
| | | EM08 | Head tosser | recessive | EM10 | Coil tail | recessive |
| | | EM13 | Myotonia | recessive | EM15 | SBS | recessive |
| | | EM15 | Nude | recessive | EM15 | Flat nose | recessive |
| | | EM20 | SBS * | recessive | EM21 | SBS | recessive |
| | | EM25 | SBS | recessive | EM26 | White spot | recessive |
| | | D2M | Short face | recessive | | | |
| E200 | 2×100 | BFM06 | High cholesterol | recessive | FDM08 | Bent tail | dominant |
| | | FDM10 | Bent tail | dominant | FDF06 | Bent tail | dominant |
| | | FDF33 | No whisker | dominant | | | |
| J200 | 2×100 | JDM02 | SBS | dominant | JDM07 | Short tail | dominant |

* SBS, small body size.

**Table 7.** The information of experimented BALB/cAnN mice

| Group | Dose (mg/kg) | Age (weeks) | Injection interval (week) | No. of males |
|---|---|---|---|---|
| Control | 0 | 12 | 1 | 11 |
| A150 | 2 × 75 | 12 | 1 | 23 |
| H150 | 2 × 75 | 12 | 1 | 19 |
| A200 | 2 × 100 | 12 | 1 | 23 |
| B200 | 2 × 100 | 10 | 1 | 55 |
| D200 | 2 × 100 | 10 | 1 | 61 |
| E200 | 2 × 100 | 12 | 1 | 19 |
| F200 | 2 × 100 | 10 | 1 | 69 |
| H200 | 2 × 100 | 12 | 1 | 10 |
| J200 | 2 × 100 | 10 | 1 | 40 |
| K200 | 2 × 100 | 10 | 1 | 25 |
| H250 | 2 × 125 | 12 | 1 | 10 |
| J250 | 2 × 125 | 10 | 1 | 10 |
| K250 | 2 × 125 | 10 | 1 | 10 |
| K300 | 2 × 150 | 10 | 1 | 10 |
| E400 | 4 × 100 | 12 | 1 | 18 |

egaly, and megalothymus. These findings are typically found in mice receiving ENU and associated with immune disorders, infertility, decreased sperm, and atrophy of the accessory reproductive glands (Davis *et al.*, 1999). Therefore, the dose of 200 mg/kg used in mutagenized males was considered to be the optimal dose for mutagenesis by inducing temporary cytotoxic effects.

ENU, a potent toxin, is well known as an efficient mutagen, producing effective mutations. ENU is also defined as genotoxicant and oncogene (Shibuya and Morimot, 1993). Genotoxicity study is one of essential tests for new chemical development (Zhang *et al.*, 2002; Jee *et al.*, 2005; Lee *et al.*, 2007). To perform an effective EUN mutagenesis screening, a dosage regimen is highly important to determine the optimal dose of EUN with a high mutagenesis rate and minimum toxic effects. For this purpose, important factors to be considered are efficient mutagenesis indicators such as no sperm and temporary infertility due to very few sperm (Justice *et al.*, 2000; Weber *et al.*, 2000; Nolan *et al.*, 1997; Kasarskis *et al.*, 1998; Moser *et al.*, 1990; Hitotsumachi *et al.*, 1985). In this study, temporary infertility and infertility within 30% were observed at 150 and 200 mg/kg until 15 weeks. Theses findings are in line with the results from the previous study in which the lethal rates of 0~100% and temporary sterile periods of 13~20 weeks were reported in ENU-treated BALB/c mice (Nolan *et al.*, 1997; Kasarskis *et al.*, 1998; Weber *et al.*, 2000). Factors that affect lethal rates and temporary sterility periods include sub-strain, age, environment, dose, injection frequency & intervals, buffer pH,

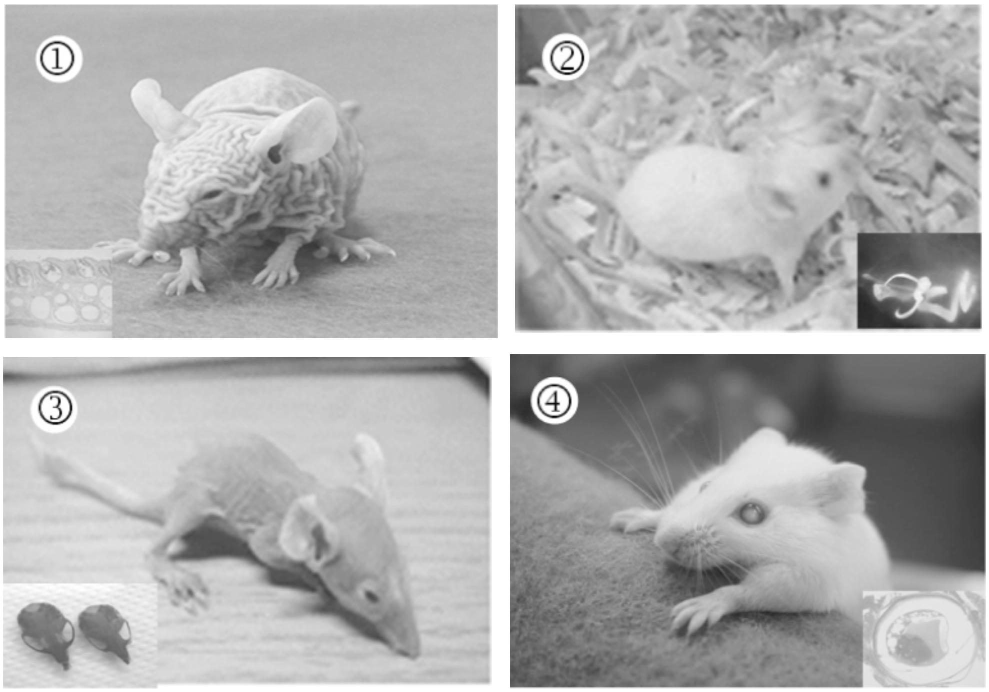

**Fig. 5.** Photographs and phenotype identification (small photographs) in the mainly mutant mice. ① Nude-like mouse at 49 days old (abnormal hair follicle cycle, cystic degeneration of piliary canal and dermal papillae, decreased number of vibrissae). ② Head tosser mouse at 28 days old (paint injection showed missing one of semicircular canals). ③ Dental aplasia mouse at 16 days old (dental aplasia in maxilla and mandible, abnormal hair follicle cycle, decreased number of vibrissae). ④ Cataract mouse at 15 days old (vacuolation in posterior part of lens).

and ENU study methods (Justice *et al.*, 2000; Shibuya and Morimoto, 1993). In this study, the lethal rate and length of sterility period were increased with increasing dose, suggesting that optimal experimental conditions and doses should be used for obtaining a high repeatability and efficiency in a research laboratory. Based on the results of the present study, the 200 mg/kg dose of ENU showing lethal rates of 10~30% and sterility period of 10~14 weeks was considered as the optimal dose for ENU mutagenesis.

Regain of fertility rate and lethal rate after regaining fertility are other mutagenesis indicators (Weber *et al.*, 2000). In general, a time period required to regain fertility in male mice that lost fertile ability due to ENU mutagenesis is approximately 10 weeks (Bode, 1984; Moser *et al.*, 1990). However, fertile ability may not be restored due to a reduction in mutation rate and decrease in spermatogenesis resulting from the depletion of spermatogonial stem cells when a sterility period lasts more than 20 weeks (Justice *et al.*, 2000). In this study, regain of fertility was found be at the appropriate level of 65% at 200 mg/kg, whereas the complete depletion of spermatogonial stem cells or very few sperm were observed at 300 mg/kg. As a next step in ENU mutagenesis, obtaining the next generation ($G_1$) mice is a priority. These mice will become the necessary founders for screening for phenodeviant and inheritance tests. In this study, $G_1$ mice at the appropriate level were obtained at 200 mg/kg, and their average litter size was decreased or slightly decreased as compared to that of the controls, corresponding with the results (3.0~5.7) from the MRC study (Nolan, personal communication). Based on these findings, the 200 mg/kg dose at which regain of fertility rate and next generation founders were stably established was considered to be the optimal dose.

ENU is highly efficient in inducing a single locus mutation as well as genome-wide mutagenesis (Hitotsumachi *et al.*, 1985), producing mutations targeting a wide-rage of diseases (Brown and Nolan 1998, Justice 1999). Therefore, it is necessary to develop an efficient phenotype screening system (Justice *et al.*, 1999). The main parameters analyzed in large-scale ENU mutagenesis laboratories include morphology, hematology & clinical biochemistry, immune, allergy, urine, behavior, blood pressure, hearing, tumorigenesis, and target disease such as development, immune, hematopoiesis, cancer, dental disorders (Justice *et al.*, 1999). The phenodeviant incidence rates for such parameters were found to be 6.4~8.3%, and abnormalities were found in behavior, skin, hair, and tail in the descending order. ECPS developed in this study was demonstrated to be a highly efficient screening method for dysmorphology and behavior abnormalities manifested at the early onset. In the present study, the phenodeviant incidence rate was as high as 8.1% (excluding biochemistry). Furthermore, phenodeviant parameters and their phenodeviant incidence rates were found to be 3.0% (tail), 0.4% (limbs), and 0.3% (craniofacial) (MRC homepage). These results strongly indicated that ECPS is efficient in screening for behavior and dysmorphology abnormalities manifested at early onset and highly effective in screening for phenodeviants of ENU mutagenesis.

With respect to the pathogenesis and mechanism of intractable diseases, mutagenized mice are intensively used as model systems due to the homologue in the human genome (Favor and Neuhauser-Klaus, 2000). A large number of ENU-mutagenized mice have been successfully produced world wide (Angeles *et al.*, 2000). However, ENU mutagenesis was rarely reported. The dominate mutation rate and recessive mutation rate in a large scale were 20~37% (Nolan *et al.*, 2000; Angles *et al.*, 2000) and 19%, respectively (Angles *et al.*, 2000). In the present study, the dominate mutation rate and recessive mutation rate were found to be 12% and 21%, respectively, suggesting a wide-range of mutations can be obtained even in a small-scale laboratory. ENU mutagenesis studies being conducted wordwide are different in terms of mutagenesis type, dose, and target disease. However, many researchers who wish to study ENU mutagenesis find it difficult to conduct related experiments due to the lack of raw data available to them. The present study was undertaken to provide useful information on ENU mutagenesis to those who wish to investigate ENU mutagenesis in the future. In conclusion, the results obtained in the present study are summarized as follows: firstly, temporary cytotoxic effects, low lethal rate and appropriate sterility period were observed at 200 mg/kg. Secondly, founders which mutagenized male mice were obtained at 200 mg/kg. Thirdly, ECPS was determined to be a highly effective screening system for ENU mutagenesis.

## ACKNOWLEDGEMENT

This study was supported by National Research Laboratory Program (2000-N-NL-01-c-207) from the Ministry of Science and Technology of Korea.
